# Morphological and molecular characterization of *Paractinolaimus uljinensis* n. sp. (Nematoda: Actinolaimidae) from Korea, with an updated compendium of the genus

**DOI:** 10.2478/jofnem-2024-0040

**Published:** 2024-10-23

**Authors:** Abraham Okki Mwamula, Yi Seul Kim, Dong Woon Lee

**Affiliations:** Research Institute of Invertebrate Vector, Kyungpook National University, Sangju 37224, Republic of Korea; Department of Entomology, Kyungpook National University, Sangju, 37224, Republic of Korea

**Keywords:** DNA barcodes, morphology, morphometrics, phylogeny, taxonomy

## Abstract

A new species of the genus *Paractinolaimus* isolated from the bark of a dead red pine tree was characterized using morphometric data and molecular DNA barcodes. *Paractinolaimus uljinensis* n. sp. was characterized by its medium sized body 2.50 to 2.98 mm long; lip region truncate, angular and offset by a depression; odontostyle 23.5 to 27.0 μm long; basal shield of pharynx present; vulval opening wide and longitudinal, positioned slightly anteriorly (V = 42.5–47.7); several advulval papillae; female tail long and filiform (324.0–435.0 μm long, c’ = 10.1–14.2); a clearly visible copulatory hump; spicules 60.0 to 70.5 μm long; 12 to 15 (mostly 12–14) large contiguous ventromedian supplements, and male tail conoid to broadly rounded. The new species was morphologically compared with *P. intermedius, P. sahandi*, *P. decraemerae, P. acutus, P. macrolaimus,* and *P. tuberculatus*. The phylogenetic relationships among species were reconstructed using 18S- and 28S-rRNA gene sequences. The phylogenies showed well-supported sister relations of *Paractinolaimus uljinensis* n. sp. with *P. sahandi, P. macrolaimus,* and *P. decraemerae*. In addition, the ITS-rRNA gene sequences of *Paractinolaimus uljinensis* n. sp. were supplied, representing the first characterization of the gene for the genus.

The order Dorylaimida Pearse, 1942 represents one of the most diversified taxonomic groups in nematode systematics. According to Peña-Santiago (2014), the taxonomy of Dorylaimida has been and is a matter of controversy. The more recent contributions to the taxonomy of the group have not offered full backing for maintaining the traditionally accepted subdivisions in superfamilies and families. Recent inferences from molecular data have also shown only distant similarity with classical morphology-based dorylaimid systematics (Holterman et al., 2008). As a result, Peña-Santiago (2014) recently amended the classification of order Dorylaimida to comprise of two suborders, i.e., Nygolaimina with four families and Dorylaimina with 14 families. The family Actinolaimidae Thorne, 1939 is one of the members of suborder Dorylaimina Pearse, 1942. Among the members of the family Actinolaimidae Thorne, 1939, the genus *Paractinolaimus* Meyl, 1957 is the richest in species and the most widespread in terms of geographical distribution, with representatives in Africa, Asia, Oceania, Europe, Russia, South and North America.

*Paractinolaimus* is mainly characterized by the presence of rasp-like denticles in the dental chamber in addition to the general characters of the “*Paractinolaimus*” genus group (Vinciguerra, 1987; Andrássy, 2012). A compendium of 32 described species of genus, with the main morphometric characters was listed by Vinciguerra et al. (2013). Since then, two new species have been described: *P. cattienus* Gusakov & Gagarin, 2015 and *P. persicus* Panahi, Shokoohi, Vinciguera, Clausi & Mashela, 2019. Two species of the genus, *P. acutus* Khan and Park, 1999 and *P. tuberculatus* Choi and Khan, 2000, have their type localities in Korea.

During a nematological survey conducted in 2024 in a natural, protected pine forest ecosystem in Korea, a population of an undescribed dorylaim belonging to *Paractinolaimus* was recovered from the bark of a dead red pine (*Pinus densiflora* for. *erecta* Uyeki) tree stand. This new species designated as *Paractinolaimus uljinensis* n. sp. is herein described based on both morphological and molecular phylogenetic comparisons. An updated compendium of the nominal species of the genus, with the main morphometric characters is also provided.

## Materials and Methods

### Nematode population and extraction

The nematode population was recovered from the bark layer of a dead (wildfire-induced mortality) red pine (*Pinus densiflora* for. *erecta*) tree taken from Geumgang pine tree forest in Uljin, Gyeongsangbuk-do Province, Republic of Korea. Nematodes were extracted from the bark cuttings using the Baermann funnel method (Baermann, 1917). Nematode specimens belonging to *Paractinolaimus* were handpicked from the resultant nematode suspension under a Nikon SMZ 1000 stereomicroscope (Nikon). Specimens were subsequently characterized based on inferences from morphometric and DNA barcode data.

## Morphological characterization

The fresh nematode specimens were heat-killed, fixed with formalin-glycerine, and processed to pure glycerin according to Seinhorst (1959) as modified by De Grisse (1969). The processed nematode specimens were mounted on permanent slides and examined under a fluorescence microscope. Measurement data and photomicrographs were taken using a Zeiss imager Z2 microscope (Carl Zeiss) fitted with Axio-vision, a Material Science Software for Research and Engineering (Carl Zeiss). Line drawings of the specimens were made under a drawing tube and redrawn using CorelDRAW® software version 24. Species identification and diagnosis was done following the diagnostic species compendia presented by Vinciguerra et al. (2013).

## Molecular characterization

Genomic DNA was extracted from morphometrically confirmed, heat-relaxed female and male specimens of *Paractinolaimus* using the DNA extraction kit WizPure™ according to Iwahori et al. (2000). Three gene fragments, 18S-rRNA gene, the D2-D3 expansion segment of 28S-rRNA gene, and the partial ITS-rRNA gene, were amplified and sequenced in this study. The nearly full-length 18S-rRNA gene was amplified as two partially overlapping fragments using two primer sets: 988F (5′-CTCAAAGATTAAGCC ATGC-3′) and 1912R (5′-TTTACGGTCAGAACTAG GG-3′), and 1813F (5′-CTGCGTGAGAGGTGAAAT-3′) and 2646R (5′-GCTACCTTGTTACGACTTTT-3′) (Holterman et al., 2006). The primer set D2A (5′-ACAAGTACCGTGAGGGAAAGTTG-3′) and D3B (5′-TCGGAAGGAACCAGCTACTA-3′) (Nunn, 1992) was used in the amplification of the D2-D3 expansion segment of 28S-rRNA gene, and the ITS-rRNA gene was amplified using S_ITS1 (5′-TTGATTACGTCCCTGCCCTTT-3′) and Vrain2R (5′-TTTCACTCGCCGTTACTAAGGGAATC-3′) (Vrain et al., 1992). A polymerase chain reaction (PCR) was performed with a PCR cycler (T100™, Bio-Rad). The thermal cycle for the primer sets D2A/D3B, 988F/1912R, and 1813F/2646R were as described by Mwamula et al. (2023), and the thermal profile for S_ITS1/Vrain2R primer set was set as detailed by Mwamula et al. (2024a). The PCR products were purified using QIAquick PCR Purification Kit (Qiagen) and quantified using a quickdrop spectrophotometer (Molecular Devices) before being directly sequenced with the primers specified above at Macrogen Inc. The edited new sequences were submitted to the GenBank database under the accession numbers PQ044581-PQ044583 (for 18S-rRNA), PQ045703-PQ045705 (for 28S-rRNA), and PQ045706-PQ045708 (for ITS-rRNA).

### Phylogenetic analysis

The new sequences (18S-rRNA, 28S-rRNA, and ITS-rRNA gene) were compared with those of related species of *Paractinolaimus*, including comparable sequences of related species from other genera published in GenBank (Holterman et al., 2006; Nedelchev et al., 2014; Vinciguerra et al., 2016; Kagoshima et al., 2019; Swart et al., 2020; Zhang et al., 2023; Mwamula et al., 2024b) using BLAST homology search program. Multiple alignments for the selected genes were built using ClustalX (Thompson et al., 1997). The sequences of *Paravulvus hartingii* (de Man, 1880) Heyns, 1968 (AY552976) and *Nygolaimus cf. parvus* Thorne, 1974 (AY552974) were used as the outgroup taxa for the 18S-rRNA gene; and *Mononchus tunbridgensis* Bastian, 1865 (AY593063) and *Anatonchus tridentatus* (de Man, 1876) De Coninck, 1939 (AY593065) were the outgroup taxa for 28S-rRNA gene. Bayesian inference (BI) of the phylogenies based on sequences of the two genes was performed using MrBayes 3.2.7 (Ronquist et al., 2012), with GTR + I + G model selected for all datasets. Bayesian inference analysis was initiated with a random starting tree and run with four chains for 1 × 106 generations. The Markov chains were sampled at intervals of 100 generations. After discarding burn-in samples, consensus trees were generated with the 50% majority rule. The generated trees were edited using FigTree v1.4.4 software. Posterior probabilities (PP) exceeding 50% are given on appropriate clades. Intraspecific and interspecific sequence distances were analyzed using PAUP* v4.0a169 (Swofford, 2003).

## Results

**Figure 1:**
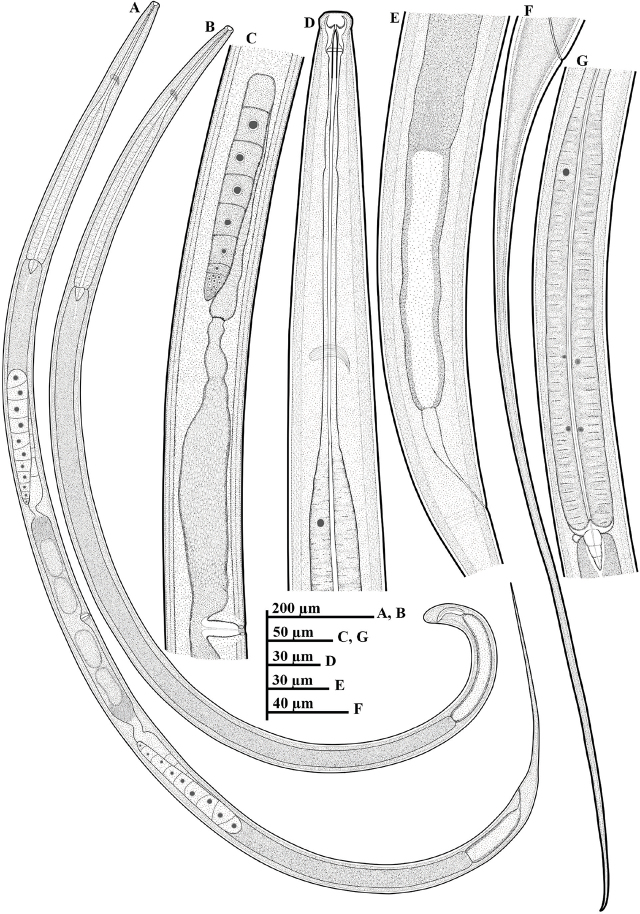
Line drawings of *Paractinolaimus uljinensis* n. sp. (A–G): A: Female whole body; B: Male whole body; C: Female reproductive system; D: Female anterior region; E: Female rectum and prerectum region; F: Female tail; G: The expanded part of pharynx (the glandularium).

**Figure 2:**
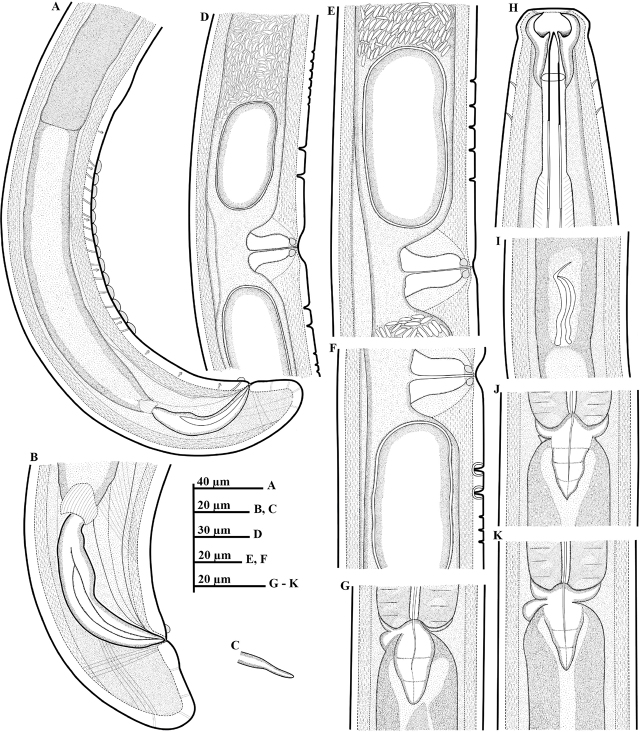
Line drawings of *Paractinolaimus uljinensis* n. sp. (A–K): A: Posterior region of male, including copulatory apparatus, the arrangement of ventromedian supplements and copulatory hump. (Arrows indicate the position of submedian papillae); B: Male tail region; C: Lateral guiding piece; D, E, F: Variation in shape and arrangement of pre/postvulval papillae; G, J, K: Variation in shape of cardia and basal shield; H: Head region; I: Junction between intestine and prerectum with a conical or tongue-like structure.

**Figure 3:**
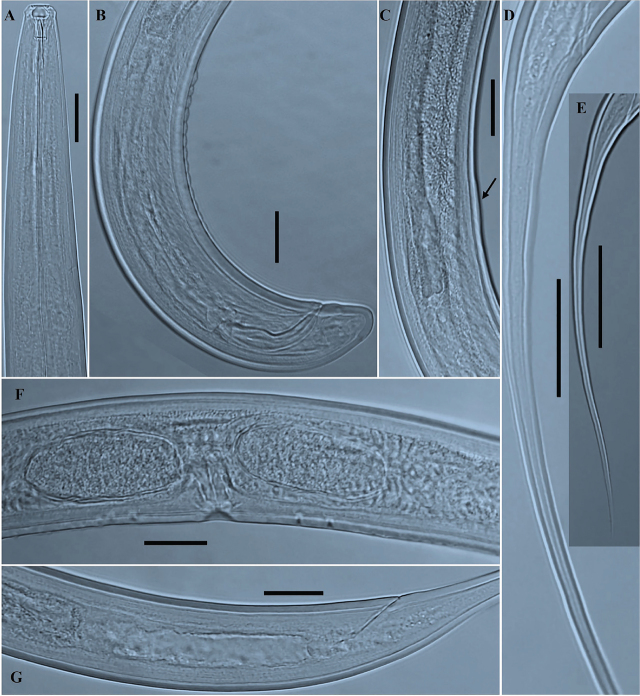
Photomicrographs of *Paractinolaimus uljinensis* n. sp. (A–G). A: Female anterior region; B: Posterior region of male, including copulatory apparatus and the arrangement of ventromedian supplements; C: Region of copulatory hump; D: Anterior and middle part of female tail; E: Whole female tail; F: Vulval region with pre/postvulval papillae conformation; G: Female posterior region, including the prerectum and rectum. (Scale bars: A, B, C, F, and G = 30 μm; D = 50 μm; E = 100 μm).

**Figure 4:**
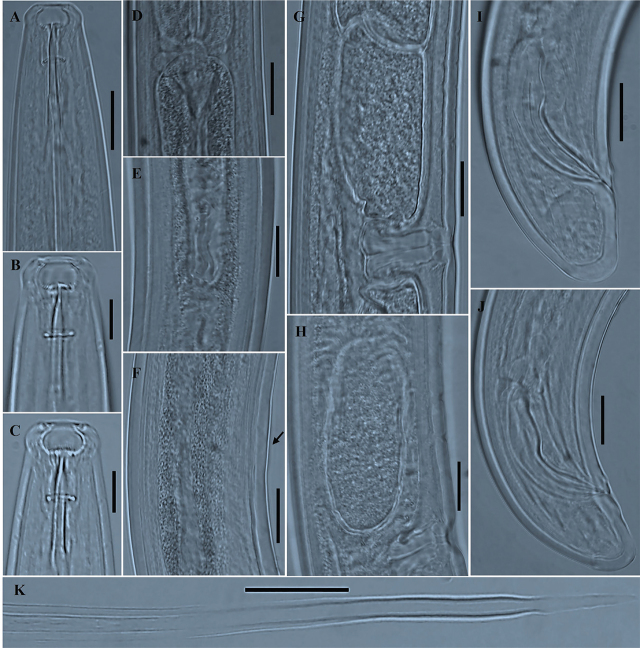
Photomicrographs of *Paractinolaimus uljinensis* n. sp. (A–K). A, B, C: Variation in female anterior region; D: Shape of cardia and basal shield; E: Junction between intestine and prerectum with a conical or tongue-like structure; F: Region of copulatory hump; G, H: Variation in shape and configuration of advulval papillae; I, J: Variation in shape of male tail; K: Posteriormost part of female tail. (Scale bars: A, D–K = 20 μm; B, C = 10 μm).

*Paractinolaimus uljinensis* n. sp. (Figs. 1, 2, 3 & 4).

### Measurements

See Table 1.

**Table 1:** Morphometrics of *Paractinolaimus uljinensis* n. sp. from Korea.

**Character**	**Holotype ♀**	**♀♀**	**♂♂**
n		30	28
L	2743.0	2764.0 ± 134.0 (2500.0–2980.0)	2449.0 ± 170.0 (2183.0–2833.0)
a	44.6	44.1 ± 2.1 (40.2–47.9)	43.1 ± 2.4 (40.2–48.9)
b	5.0	4.9 ± 0.2 (4.5–5.2)	4.4 ± 0.3 (3.8–4.9)
c	7.5	7.7 ± 0.4 (6.9–8.5)	103.7 ± 9.4 (82.9–121.4)
c’	12.2	11.7 ± 0.9 (10.1–14.2)	0.7 ± 0.1 (0.6–0.9)
V	45.6	45.5 ± 1.3 (42.5–47.7)	_
G1%	17.6	16.6 ± 1.6 (13.7–20.7)	_
G2%	18.0	17.9 ± 1.5 (14.6–21.5)	_
Lip height	7.5	7.6 ± 0.4 (7.0–8.5)	7.6 ± 0.5 (7.0–8.5)
Lip diam.	19.5	19.6 ± 0.6 (18.5–20.5)	19.6 ± 0.5 (19.0–20.5)
Anterior to guiding ring	19.5	19.2 ± 0.7 (17.5–20.5)	19.0 ± 0.5 (17.5–20.0)
Odontostyle	26.5	25.2 ± 1.0 (23.5–27.0)	25.1 ± 0.8 (23.5–27.0)
Odontophore	24.0	23.4 ± 0.9 (22.0–25.0)	23.3 ± 0.8 (22.0–25.0)
Total spear	50.5	48.5 ± 1.5 (45.5–51.0)	48.3 ± 1.3 (46.5–52.0)
Anterior to nerve ring	156.5	158.7 ± 6.0 (147.0–173.0)	158.7 ± 6.0 (146.0–172.0)
Pharynx length	546.0	562.4 ± 20.7 (502.0–598.0)	562.9 ± 19.2 (518.0–597.0)
Glandularium length	329.0	341.9 ± 15.5 (307.5–368.0)	348.1 ± 16.4 (313.5–384.5)
Cardia length	22.0	24.7 ± 3.6 (17.5–30.5)	24.5 ± 3.0 (19.0–29.5)
Maximum body diam.	61.5	62.8 ± 3.1 (55.5–67.5)	56.8 ± 2.0 (53.5–61.5)
Prerectum	116.0	122.9 ± 18.7 (86.5–169.5)	200.3 ± 20.1 (174.0–246.5)
Rectum	49.5	48.6 ± 5.2 (40.0–56.0)	58.6 ± 2.5 (52.5–63.0)
Anal / cloacal body diam.	30.0	31.0 ± 1.6 (26.5–33.0)	33.1 ± 1.6 (30.0–36.0)
Tail length	365.0	361.3 ± 25.8 (324.0–435.0)	23.8 ± 2.0 (21.0–29.0)
Spicules	_	_	64.5 ± 2.5 (60.0–70.5)
No. of ventromedian supplements	_	_	12–15

### Description

#### Female (n = 30)

General habitus curved ventrally to C-shape when heat-killed and fixed. Cuticle 2.0 to 3.0 μm thick at level of guiding ring, 3.0 to 4.0 at level of cardia, 4.0 to 5.0 μm thick at mid-body, and 4.5 to 6.0 μm at anal level. Lateral chords occupying about one-fifth of corresponding body diameter. Lip region 2.3 to 2.8 times as wide as high, anteriorly truncate, with mainly angular sides, appearing rounded in a few specimens, offset by a depression. Vestibular ring corrugated. Cheilostome well sclerotized with four onchia and several pointed denticles. Amphids stirrup-shaped, with a wide opening, occupying 45% to 48% of lip region diameter. Odontostyle dorylaimoid, massive, *ca* 1.2 to 1.4 times the lip region diameter. Odontostyle aperture occupying 32% to 44% of odontostyle length. Odontophore simple, rod-like. Guiding ring “double,” located at 17.5 to 20.5 μm or one lip region diameter from anterior end. Two body pores present both dorsally and ventrally at the level of odontostyle: first pore located 0 to 5.0 μm posterior to the level of guiding ring, second pore located 5.0 to 10.0 μm from first pore. Pharynx muscular, consisting of a slender anterior region, gradually increasing in diameter. Nerve ring encircling the slender part of corpus at 147.0 to 173.0 μm or 26% to 32% of the total pharynx length from anterior end. The glandularium (the expanded part of the pharynx) beginning as a gradual expansion at 30.0 to 48.0 μm behind the nerve ring or at 36.6% to 43.7% of the total pharynx length from anterior end, attaining full expansion at 97.5 to 124.0 μm from the nerve ring, and occupying 56.3% to 63.4% of the total neck length. Location of pharyngeal gland nuclei with respect to total pharynx length (%): D = 49.2 to 55.5, AS1 = 74.0 to 76.7, AS2 = 75.0 to 78.5, and PS = 85.1 to 88.3 (for formulae and terminology, see Loof and Coomans (1970), and Andrássy (1998), respectively). Basal shield of pharynx present often with extended bulges at pharynx-intestine junction. Cardia variable in length, 17.5 to 30.5 μm long, conoid, projecting into the intestine. Reproductive system didelphic amphidelphic, well-developed, with anterior and posterior branches of nearly equal length. Each branch basically arranged from a fully developed and reflexed ovary to gonoduct. Oviduct outstretched, oviduct-uterus junction with a sphincter. Uterus with a narrow distal part and an enlarged proximal part, filled with abundant well-developed, sausage to spindle-shaped sperm. Vagina perpendicular to body axis, occupying nearly half of corresponding body diameter (44.0%–49.0%). *Pars proximalis vaginae* cylindroid, 19.5 to 26.5 μm long and 12.0 to 15.0 μm wide; *pars refringens vaginae* with two irregularly ovoid pieces in lateral view, 3.5 to 7.0 μm deep and 3.0 to 5.5 μm wide, with a combined width of 10.5 to 14.5 μm; and *pars distalis vaginae* reduced, 2.5 to 4.0 μm long. Vulva appearing wide and longitudinal in profile. Several pre- and postvulval papillae present in all specimens examined, the number generally varying from three to nine on both sides, (i.e., pre/postvulval papillae 3–9, 3–9), rarely in two pre- and two postvulval configurations (observed in only one specimen). In some specimens, the first two pre- and postvulval papillae appeared more prominent, followed by several small papillae without visible innervations into the inner layer of the cuticle. An anteriorly directed conical or tongue-like projection, 20.0 to 45.0 μm long present at the intestine-prerectum junction in several specimens examined. Rectum and prerectum 1.3 to 1.8 and 3.1 to 5.6 times anal body diameter long, respectively. Tail generally long, curved ventrad or straight. Anterior part (29.0–47.5 μm from anal opening) convex conoid, continuing gradually, and tapering to a straight middle part, becoming filiform posteriorly, with a finely rounded or pointed tail terminus. Total tail length 10.1 to 14.2 times anal body diameter.

#### Male (n = 28)

Generally as abundant as females. General morphology similar to that of females except for sexual characters and conoid to a broadly rounded tail. Body generally curved ventrally to a J-shape when heat-killed and fixed. Genital system diorchic, testes symmetrical, well-developed, with well-developed sausage to spindle-shaped sperms, 6.0 to 8.0 μm long. Spicules massive, ventrally curved, 60.0 to 70.5 μm long, or 1.8 to 2.1 times cloacal body diameter. Lateral guiding pieces 17.0 to 21.0 μm long. Supplements consisting of a precloacal pair located at 2.0 to 5.0 μm from cloacal opening and a series of 12 to 15 (mostly 12–14 in number; 15 observed in only one of the specimens examined) large contiguous ventromedian supplements. A clearly visible copulatory hump present in all specimens examined, 18.0 to 28.5 μm long, located 31.0 to 48.0 μm anterior to the anteriormost ventromedian supplement. Ventromedian supplements starting at 170.0 to 211.0 μm from cloacal opening, spaced 6.0 to 8.5 μm apart (measured between ducts), occupying 90.0 to 125.0 μm of the ventral contour length, posteriormost ventromedian (nearest to cloacal opening) located at 74.0 to 92.0 μm from cloacal opening. Eight ventrolateral (submedian) papillae present in the precaudal region, arranged in a sequence: four spaced within the range of the supplements, and one anterior and three posterior to the range of supplements. Intestine-prerectum junction with an anteriorly directed conical or tongue-like projection in some specimens. Position of intestine-prerectum junction in relation to supplements variable, located mostly beyond the range of supplements (0–70 μm), rarely terminating within the range of supplements (0–19.0 μm from anteriormost supplement) (the latter observed in only two specimens). Prerectum and rectum 5.0 to 7.6 and 1.5 to 2.0 times cloacal body diameter long, respectively. Tail shorter than cloacal body diameter (0.6–0.9 times cloacal body diameter), conoid to broadly rounded, with four distinct pairs of caudal pores.

### Diagnosis and relationships

*Paractinolaimus uljinensis* n. sp. is characterized by having a medium sized body 2.50 to 2.98 mm long; lip region truncate, angular and offset by a depression; odontostyle 23.5 to 27.0 μm long; basal shield of pharynx present; well-developed, didelphic-amphidelphic female reproductive system; vulval opening wide and longitudinal in profile, positioned slightly anteriorly (V = 42.5–47.7); several advulval papillae; intestine-prerectum junction with an anteriorly directed conical or tongue-like projection; a long female tail (324.0–435.0 μm long, c’ = 10.1–14.2), tapering to a straight middle part, becoming filiform posteriorly, with a finely rounded or pointed tail terminus; a clearly visible copulatory hump; spicules 60.0 to 70.5 μm long; 12 to 15 (mostly 12–14) large contiguous ventromedian supplements and male tail conoid to broadly rounded.

By having advulval papillae, *Paractinolaimus uljinensis* n. sp. is very close to *P. intermedius* Altherr, 1968; *P. sahandi* Pedram, Niknam, Vinciguerra, Ye & Robbins, 2011; *P. decraemerae* Pedram, Niknam, Vinciguerra, Ye & Robbins, 2010; *P. acutus* Khan & Park, 1999; and *P. macrolaimus* (De Man, 1880) Andrássy, 1964. It differs from all, except *P. sahandi*, by the presence of a higher number of advulval papillae in front and behind the vulva, including the presence of several small papillae without visible innervations in individuals with a few prominent papillae (this configuration is evident in only *P. sahandi*). Additionally, *Paractinolaimus uljinensis* n. sp. differs from the closest *P. intermedius* by its truncate lip region, with angular lips set off from the body by a depression *vs* lips not angular, rounded, with papillae slightly protruding from their profile, continuous with body or hardly separated by a very slight depression, long female tail (c = 6.9–8.5 *vs* 13–17; c’ = 10.1–14.2 *vs* 6–7), a more anteriorly positioned vulva (V = 42.5–47.7 *vs* 46–54), and a relatively lower number of contiguous ventromedian supplements (12–15) *vs* relatively higher number of spaced ventromedian supplements (14–19); from *P. sahandi* by body length (2.5–3.0 mm *vs* 3.5–4.7 mm in females, and 2.2–2.8 mm *vs* 3.2–4.4 mm in males), a ratio (40.2–47.9 *vs* 74.5–88.5), long tail (324.0–435.0 *μm vs* 198.5–270.0 μm; c = 6.9–8.5 *vs* 16.2–22.0, c’ = 10.1–14.2 *vs* 4.5–7.5), a relatively shorter odontostyle (23.5–27.0 *vs* 27.0–32.5), a more anteriorly positioned vulva (V = 42.5–47.7 *vs* 47–54), and the presence of a copulatory hump *vs* no copulatory hump; from *P. decraemerae* by the angular lips *vs* rounded, long tail (324.0–435.0 *μm vs* 200–238 μm ; c = 6.9–8.5 *vs* 13.3–16.0, c’ = 10.1–14.2 *vs* 5.5–6.7), males as abundant as females *vs* very rare, shorter spicules (60.0–70.5 μm *vs* 76.5 μm), lower number of supplements (12–15 *vs* 16), and the presence of a copulatory hump *vs* no copulatory hump; from *P. acutus* by long body (2.5–3.0 mm *vs* 2.1–2.4 mm), angular lips offset by a depression *vs* rounded and continuous with body, longer tail (324.0–435.0 μm *vs* 285.0–336.0) terminating in a finely rounded or pointed tip *vs* ending in a distinct acute tip, vulva shape (longitudinally oval, situated in a small depression in *P. acutus*), and males as abundant as females *vs* no males.

Regarding comparison with *P. macrolaimus, Paractinolaimus uljinensis* n. sp. is distinctly different from *P. macrolaimus* type specimens in almost all attributes: short body (2.5–3.0 mm *vs* 3.7–4.5 mm), presence of several advulval papillae *vs* absent, a ratio (40.2–47.9 *vs* 50), c ratio (6.9–8.5 *vs* 11.1–11.8), lower number of ventromedian supplements (12–15 *vs* 16–24), and the presence of a copulatory hump *vs* absent. However, the new species is comparable to the morphologically divergent African population that was described by Andrássy (1964), and Andrássy (2009) (see Vinciguerra et al. (2013), and Pedram et al. (2011)). Despite the discrepancies in morphological details of this population in relation to the morphology of the type specimens of *P. macrolaimus* (De Man, 1880), including the subsequent redescriptions by Loof (1961), *Paractinolaimus uljinensis* n. sp. still differs from this population by its shorter odontostyle (23.5–27.0 *vs* 27.0–30.0), advulval cuticular configurations (several prominent papillae, including several small papillae without visible innervations in individuals with few prominent papillae), lower number of supplements (12–15 *vs* 16–19), and the presence of a copulatory hump.

Also, based on morphometric measurements, *Paractinolaimus uljinensis* n. sp. comes close to *P. tuberculatus* Choi & Khan, 2000. However, *Paractinolaimus uljinensis* n. sp. can be qualitatively differentiated from *P. tuberculatus* by its angular lips (set off by a depression *vs* rounded and continuous or almost continuous), presence of several advulval papillae *vs* absent, vulva longitudinal *vs* transverse slit, presence of a copulatory hump *vs* absent, ventromedian supplements 12 to 15, contiguous in arrangement *vs* 15 to 19 (regularly spaced), and the presence of a tubercle-like structure in the lumen of pharynx base in *P. tuberculatus,* hence its species epithet name.

### Type habitat and locality

The nematode population was recovered from the bark of a dead red pine (*Pinus densiflora* for. *erecta*) tree stand from Geumgang pine tree forest in Uljin, Gyeongsangbuk-do Province. GPS coordinates: 37°01′50″N, 129°22′83″E).

### Type material

Holotype female, 25 female, and 24 male paratypes were deposited in the Nematode Collection of Kyungpook National University (KNU), Republic of Korea, and five female and four male paratypes were deposited in the Nematode Collection of Ghent University Museum in Belgium.

### Etymology

*Paractinolaimus uljinensis* n. sp. was isolated from a red pine tree stand from Geumgang pine tree forest in Uljin, Gyeongsangbuk-do Province. Thus, the species’ epithet *uljinensis* is derived from the locality of its first description, i.e., Uljin.

### Molecular characterization and phylogenetic relationships

The amplification of the nearly full-length 18S-rRNA, the D2-D3 expansion segment of 28S-rRNA gene and ITS1-5.8S-ITS2 of rRNA yielded fragments of approximately 1700 bp, 800 bp, and 1200 bp, respectively. No intraspecific sequence variation was recorded in the three newly obtained 18S-rRNA gene partial sequences (PQ044581–PQ044583). In the 18S-rRNA gene phylogeny, *Paractinolaimus uljinensis* n. sp. sequences were grouped in a supported subclade with the closely homologous sequences of *Paractinolaimus sahandi* (GU178030), *Trachactinolaimus nanjingensis* (ON054242 and ON054246), *Paractinolaimus* sp. (OQ682610), *Enchodelus cf nepalensis* (KJ636402), *Paractinolaimus macrolaimus* (AY284826, AY993978, and KJ636378), *Paractinolaimus decraemerae* (GU446710), *Prodorylaimus* sp. (EF207246), *Dorylaimus stagnalis* (AY284776), and *Mesodorylaimus* sp. (MG921255), differing by 10 bp (0.8%), 12 to 13 bp (0.8%), 13 bp (0.8%), 14 bp (0.8%), 14 to 16 bp (0.9%), 15 bp (0.9%), 21 bp (1.2%), 21 to 22 bp (1.3%), and 21 to 22 bp (1.3%), respectively.

**Figure 5:**
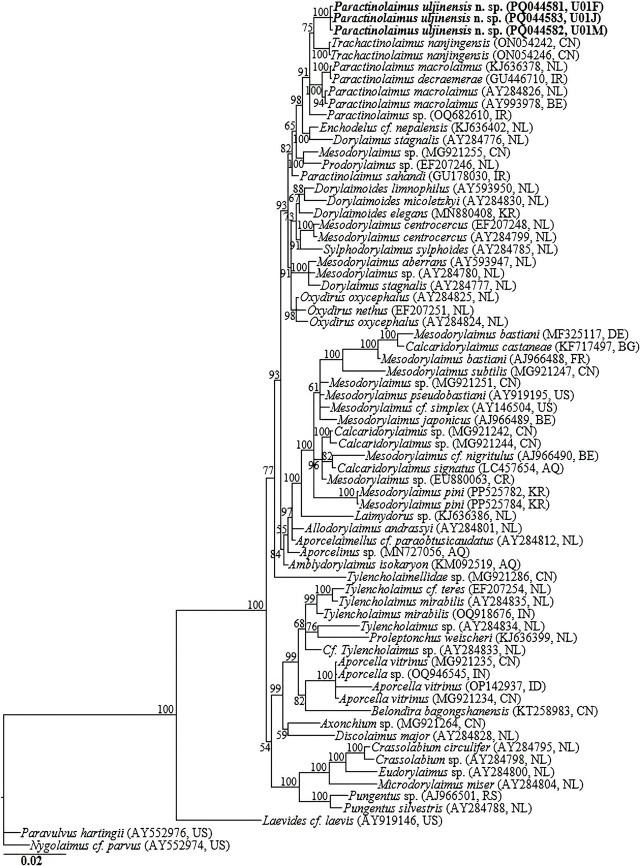
Bayesian tree inferred under the GTR + I + G model from 18S-rRNA gene sequences of Dorylaimid species. Posterior probability values exceeding 50% are given on appropriate clades. The studied population is indicated in bold text. Outgroup taxa: *Paravulvus hartingii* and *Nygolaimus cf. parvus*.

**Figure 6:**
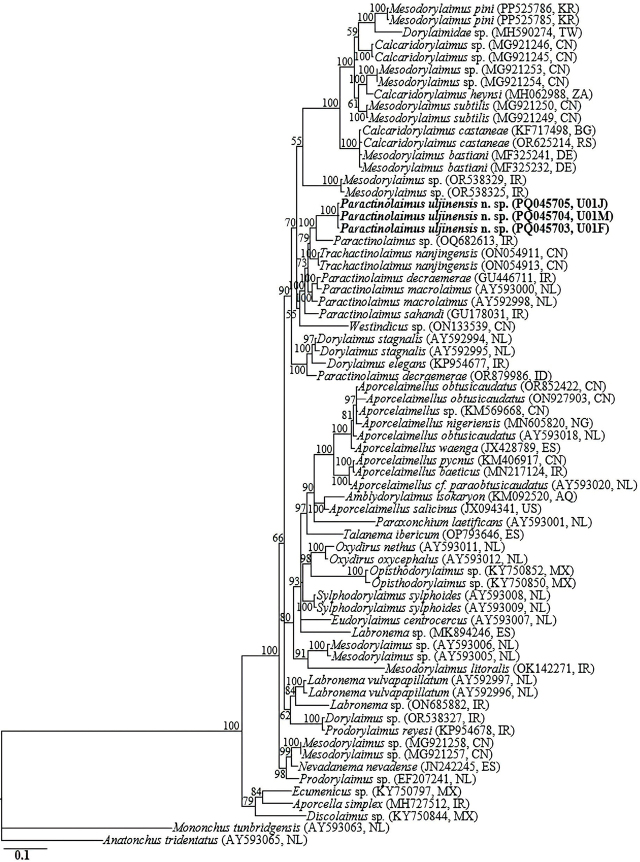
Bayesian tree inferred under the GTR + I + G model from LSU D2-D3 partial sequences of Dorylaimid species. Posterior probability values exceeding 50% are given on appropriate clades. The studied population is indicated in bold text. Outgroup taxa: *Mononchus tunbridgensis* and *Anatonchus tridentatus*.

The three D2-D3 sequences of *Paractinolaimus uljinensis* n. sp. (PQ045703–PQ045705) were also identical, with no intraspecific sequence variation (0.0%). And, based on the more informative 28S-rRNA gene phylogeny, the sequences of *Paractinolaimus uljinensis* n. sp. were grouped in a well-supported subclade (PP = 100%) with the sequences of *Trachactinolaimus nanjingensis* (ON054911 and ON054913), *Paractinolaimus* sp. (OQ682613), *Paractinolaimus decraemerae* (GU446711), *Paractinolaimus macrolaimus* (AY592998 and AY593000), and *Paractinolaimus sahandi* (GU178031), differing by 44 to 46 bp (5.6–6.0%), 47 bp (6.3%), 45 to 51 bp (6.0–6.3%), 46 to 56 bp (6.1–7.0%), and 53 to 59 bp (7.0–7.3%), respectively. The ITS1-5.8S-ITS2 of rRNA gene sequences of *Paractinolaimus uljinensis* n. sp. represent the first characterization of the gene for the genus, and therefore, there were no definitive ITS-rRNA gene sequences for *Paractinolaimus* in GenBank for comparison. However, the three generated sequences (PQ045706-PQ045708) showed relative homology with ITS-rRNA gene sequences of *Trachactinolaimus* sp. available in GenBank (percentage identity of 89.6%, but with a low query coverage of 43%). 70 18S-rRNA and 69 28S-rRNA gene sequences from member species of *Paractinolaimus,* and other related genera, including the newly obtained sequences and outgroup taxa, constituted the sequence dataset for phylogenetic analyses. Phylogenetic relationships, as inferred from Bayesian analysis of the dataset with GTR + I + G substitution model, are shown in Figs. 5 and 6.

## Discussion

Analyses of 18S-rRNA gene and especially the more informative D2-D3 expansion of 28S-rRNA gene suggest that *Paractinolaimus uljinensis* n. sp. is genetically distinct from the few available *Paractinolaimus* gene sequences as represented by the Bayesian trees. The phylogenetic inferences suggest that *Paractinolaimus uljinensis* n. sp. is a sister species to the morphologically close *P. decraemerae* and *P. sahandi.* As already noted above and discussed by Thorne (1939), Pedram et al. (2011), and Vinciguerra et al. (2013), there appears to be an overestimation of the intraspecific variation within the type species *P. macrolaimus.* The African population reported by Andrássy (1964) as a representative of *P. macrolaimus* might be a representative of an undescribed species of the genus. In the original description and subsequent redescription of the type species by De Man (1880) and Loof (1961), the presence of advulval papillae was not reported. However, this character was recorded in the African population (Andrássy, 1964). Based on the characters of this divergent population, Andrássy (2009) synonymized *P. intermedius* with *P. macrolaimus.* However, *P. intermedius* distinctly differs from the African population in various qualitative characters (see Altherr, 1968). We therefore agree with Vinciguerra et al. (2013) that the widely reported populations identified as *P. macrolaimus,* but with phenotypic plasticity in various morphological characters reveal the existence of a *macrolaimus* or an *intermedius* group of species. Additionally, the genus has been amended by various taxonomists and some of the species have been transferred to other genera. Herein, an updated compendium of the nominal species, with their main morphometric characters, is provided (Table 2).

**Table 2:** Main morphometric characters of *Paractinolaimus* species (updated from Vinciguerra et al. (2013)). Measurements in μm, except L in mm. (* measurements obtained from original drawings).

**Species**	**Sex**	**L**	**a**	**c**	**c’**	**V**	**Odont.**	**Pre/postvul. papillae**	**Spic.**	**Ventr. suppl.**	**Reference**
*acutus*	♀	2.1–2.4	36–43	10–12	10–12	44–46	25–27	1, 2			Khan and Park (1999)
*aruprus*	♀	2.25–2.40	30–32	8	8.1	45–48	23–26	none			Khan et al. (1994)
*baldus*	♀	2.6	46	11	8.7*	52	27	none			Thorne (1967)
	♂	2.9	49	84	0.7*		27			10–11	
*cattienus*	♀	2.38–2.43	40–51	8.2–10.1	9.6–12.7	47.8–48.6	18–19	none			Gusakov and Gagarin (2015)
	♂	2.22	39	148	0.6		19		48	12	
*chandicus*	♀	2.1–2.3	44–54	14–17	5*	51–52	27–29	none			Khan and Jairajpuri (1994)
	♂	2.1	43	88	0.9*		27		60	11	
*chiki*	♀	2.6–2.9	63–64	15–17	6.5–6.8	48–49	20–21	none			Dhanam et al. (1994)
*decraemerae*	♀	2.8–3.2	36–48	13–16	5.5–6.7	46–51	25–29	1, 1			Pedram et al. (2010)
	♂	2.1	32	68	0.6		27		76	16	
*dhanachandi*	♀	1.9	37	10	6.7	52	23				Khan and Jairajpuri (1994)
*elongatus*	♀	3.1–3.4	62–65	16–17	5.3–5.5	49–53	31–33	none			Khan and Jairajpuri (1994)
	♂	3–3.1	56–68	116–118	0.6–0.7		31–33		58–62	18–21	
*filipjevi*	♀	3.3	43	6.8	12.1*	45	35				Schneider (1935)
*girini*	♀	2–2.3	28–29	18.7–22.4	3	54–56	24				Sukul (1967)
	♂	2.1	28	71			24			13	
*indicus*	♀	1.5–1.9	31–37	15–37	1.4–5.5	53–56	20–22	none			Khan and Ganguly (1988)
*intermedius*	♀	2.5–2.8	36–53	13–17	6–7	46–54	27	present			Altherr (1968)
	♂	2.6–3.6	36–60	70–115					63–65	14–19	
*ishibashi*	♀	3.0–3.3	51–55	8.3–9.9	6.2–6.8	49–52	27–28	1, 1			Khan et al. (2008)
*longidrilus*	♀	3–3.7	35–45	10–14	5.3–7.1	46–50	32–33	1–2, 1–2			Eveleigh (1982)
	♂	3.5	46	91	0.8		33		83	19	
*macrolaimus*	♀	4.5	50	11.5	47			none			de Man (1880)
	♂	3.7	50	85						16–24	
	♀	3.1–3.3	40–42	11.1–11.8		46–51		none			Loof (1961)
	♀	2.7–4.3	42–60	9.0–14	8–12	44–50	27–30	present			Andrássy (2009)
	♂	2.5–3.7	46–57	90–120			27–30			16–19	
*magistris*	♀	1.9	34	51–53	1–1.1	53–54	28–30	none			Vinciguerra et al. (2013)
	♂	2–2.3	42–45	60–76	0.8		31–35		53–60	14–15	
*micoletzkyi*	♀	1.9	29	24	2.3	54	28	none			Coomans et al. (1990)
	♂	2.4	40	82	0.7		29		69	11	
*microdentatus*	♀	3.4–3.5	43–53	12–14	7.6–8.5	51–53	31–35	none			Andrássy (1964)
	♂	2.9–3.4	38–59	96–100			31–35		67–70	18–24	
*occalescens*	♀	2.6	37	10	6*	51					Schneider (1937)
	♂	1.9	27	64	0.8					9	
*pachydermis*	♀	2.1	33–34	7.6–8	8–8.5	44–45	26–27	0, 1			Khan and Araki (2001)
*parietinus*	♀	1.8–2.4	27–38	9.0–14	5.8*	49–54	25	none			Eroshenko (1977)
	♂	1.7–2.4	25–34	50–74					70	16	
*persicus*	♀	2.3–2.6	32–35.5	11.7–16.9	3.3–5.9	47.6–51.6	25–28	none			Panahi et al. (2019)
	♂	2.1–2.68	40.3	88.3	0.7		28–29		71–75.5	17–18	
*rafiqi*	♀	4.4–5.3	72–89	20–27	4.4–6.5	50–52	33–35	2–3, 2–3			Khan and Jairajpuri (1998)
	♂	5.0–5.2	85–87	198–209	0.9*		32–33		60*	21	
*robustus*	♀	3	43	11	11*	50	23	none			Thorne (1967)
	♂	2.8	47	71			23			10	
*sahandi*	♀	3.5–4.7	74–88	16–22	4.5–7.5	47–54	27–32	1–4, 1–3			Pedram et al. (2011)
	♂	3.2–4.4	70–93	116–166	0.6–0.7		27–31		62–81	15–17	
*shamimi*	♀	1.4–1.5	46–52	3.7–4.2	19–20	41–42	12–15	none			Gantait et al. (2006)
*spanithelus*	♀	2.8–3.9	35–53	13–18	5.0–6.3	45–49	29–33	0, 0–1			Eveleigh (1982)
	♂	3.4	36	76	1		30		67	12	
*tuberculatus*	♀	2.3–2.5	39–44	7.2–8.8	8.8–10.2	46–47	24–28	none			Choi and Khan (2000)
	♂	1.9–2.2	34–39	68–78	0.7*		24–27		66–69	15–19	
*uljinensis n. sp.*	♀	2.5–3.0	40.2–47.9	6.9–8.5	10.1–14.2	42.5–47.7	23.5–27.0	3–9, 3–9			This study
	♂	2.2–2.8	40.2–48.9	82.9–121.4	0.6–0.9		23.5–27.0		60–70.5	12–15	
*vigor*	♀	2	34	14		53	28	none			Thorne (1967)
*vulvapapillatus*	♀	1.6	34	9	6.9	64	22	3, 3			Khan et al. (1994)
	♂	1.4–1.5	28–32	57–63	0.8		19–20		42–45	9	
*xosorum*	♀	1.66	29	18	3.2*	52	13				Heyns and Argo (1969)

Abbreviations: Odont. (Odontostyle); Pre/postvul. Papillae (Pre/postvulval Papillae); Spic. (Spicules); Ventr. suppl. (Ventromedian supplements).

Integrative taxonomy considering both morphometrics and DNA barcodes provide a better supported approach in delineating cryptic nematode species (Subbotin, 2015; Mwamula et al., 2024b). The available DNA barcodes, including the newly obtained sequences in the current study, support the genetic distinctness and confirm the taxonomic positions of some of the species that are closely related to *P. macrolaimus*. It is imperative that reference DNA barcodes from type material of *P. intermedius,* or from type locality-collected specimens, be obtained as this will resolve the taxonomic position of other cryptic populations and supplement the current generic compendium.
